# Use of audio-visual aids and case studies to enhance understanding of family medicine among medical students

**DOI:** 10.4102/phcfm.v16i1.4278

**Published:** 2024-02-14

**Authors:** Stephen T. Engmann

**Affiliations:** 1 Family Medicine Unit, Manna Mission Hospital, Accra, Ghana; 2 Family Health Medical School, Family Health University College, Accra, Ghana

**Keywords:** medical students, learning aids, case study, family medicine, education

## Abstract

Teaching family medicine to medical students is primarily intended to aid in their understanding of the distinctive and significant role that the speciality performs within the healthcare system. Improving medical students’ comprehension of family medicine may have an impact on their decision to pursue family medicine as a speciality. It is important to use innovative evidence-based teaching and learning strategies and ensure that medical students receive extra learning opportunities in family medicine. This is a short report highlighting the use of audio-visual aids and case studies to enhance the understanding of family medicine principles among undergraduate medical students attending a private university in Ghana.

**Contribution:**

This short report offers family medicine teachers and educators at the undergraduate level an example of how to apply audio-visual aids and case studies to enhance the understanding of family medicine principles among students. The report contributes to the growth of family medicine as a speciality within the African context.

## Background

In sub-Saharan Africa (SSA), family medicine is still developing and is responsive to the unique requirements of the communities it serves as well as organisational structures and health system designs.^1^ Helping medical students to comprehend the unique and significant role family medicine plays within the healthcare system is the primary objective of teaching family medicine.^2^ A study in Ghana among medical students reported a good familiarity with family medicine, yet few of them wish to specialise in it because of an inadequate understanding of the field.^3,4^ The medical students lacked the knowledge necessary to influence their decision to pursue family medicine as a speciality.^4^ It was, therefore, necessary to provide medical students with early exposure to the principles of family medicine.^3,4,5^ In most undergraduate curricula, there is little room for family medicine instruction and so more needs to be done to promote inclusivity within this field.^6^

Medical education should be flexible enough to accept and make use of multidisciplinary teaching paradigms, beginning in the preclinical years.^7^ The core values of family medicine among students and trainees can be strengthened by encouraging one another and sharing and using innovative educational methods.^8^ These core principles of family medicine that students are exposed to include compassionate care; a holistic approach to care focusing on the whole person, the family and the community; continuity and coordination of care; a trusted patient–physician relationship and lifelong learning.^2^ Following this background, this short report highlights the use of audio-visual aids and case studies to enhance the understanding of family medicine principles among undergraduate medical students in a private university college in Ghana.

## Experience of introducing medical students to family medicine

This short report is an account of the teaching and learning activities in family medicine undertaken by the author at the Family Health Medical School of the Family Health University College located in the Greater Accra region of Ghana. Family Health Medical School is a private medical school affiliated to the University of Ghana.^9^ As part of the medical school curriculum, students are exposed to family medicine through lectures during their coordinated course in medicine and surgery in the fourth year of their medical training. During this period, the students are introduced to the concepts, principles and core values of family medicine. The author has been a course instructor or lecturer for family medicine in the school since the year 2021. The topics students are taught to introduce them to family medicine include Patient–Centred Care and the Family Physician, Family Dynamics in Health and Disease, General Systems Approach to Health and Disease, Coordination in Patient Care, Communication in Family Medicine and the Role of Spirituality in Patient Care. During these lecture sessions, audio-visual aids and case studies are employed to enhance the understanding of students in the different aspects of the field of family medicine.

### Use of audio-visual aids

An audio-visual aid has been defined as any tool that expands on what a person already knows from reading by using sight and sound.^10^ When there is a clear connection between the visual aids and the course material, students find the sessions relevant and beneficial.^10,11^ Furthermore, using audio-visual aids in the classroom can help teachers improve lesson ideas and give students new ways to digest knowledge.^11^ In the use of these aids by the author, videos chosen for this purpose are usually concise with a direct message about the topic and are of short duration to avoid the boredom that can arise from watching a long video. The videos serving as audio-visual aids are downloaded from youtube and are played and projected on a screen through a projector. Students are usually encouraged to share their reflections on the video with the class after watching the video. Table 1^12,13,14,15^ shows the video description and web links to some of the videos used for teaching.

**TABLE 1:** Video description and web links to sample videos used for teaching medical students.

Lecture topic	Video description /documentary	Youtube link to video
1. Introduction to family medicine and patient-centred care	Introduction to FamilyMedicine and Its Principles What does a familymedicine doctor do?	https://www.youtube.com/watch?v=J3Q9jMgWSPk https://www.youtube.com/watch?v=RD1rLgoUuZ4
2. Management of the chronically ill patient	The Role of FamilyPhysicians in Managing Chronic Diseases	https://www.youtube.com/watch?v=kzqpO9V73rU
3. Role of spirituality in patient care	Dr. Eduardo Bruera – The Value of Spirituality inPatient Care	https://www.youtube.com/watch?v=BAe-QZAQ7y0

*Source:* Please see the full reference list of the article Engmann ST. Use of audio-visual aids and case studies to enhance understanding of family medicine among medical students. Afr J Prm Health Care Fam Med. 2024;16(1), a4278 https://doi.org/10.4102/phcfm.v16i1.4278, for more information

### Use of case studies

Findings from a meta-analysis on the effectiveness of case-based learning suggests that it is an active teaching strategy when applied in pharmacy and medical education.^16^ Case studies are frequently used to enhance and supplement didactic instructional content, and they can be customised for a range of teaching contexts.^17,18^ The case study approach encourages students to get the theoretical knowledge and practical skills necessary to carry out their professional duties.^18^ The use of case studies has been demonstrated to boost students’ interest in contemporary medical issues and solutions to problems in the international medical community.^18^ For example, in the teaching of the students at the Family Health Medical School, one of the areas where case studies were applied was in the coordination of care. Separate case studies are presented to students to demonstrate care coordination as well as fragmented care. ‘Ms. G: A case study in fragmented care’ is an example of a case study used during lectures.^19^ This case study serves as an example of the dangers of fragmented care, which involves numerous clinicians who are not efficiently exchanging information. ‘Ms. H: A case study in coordinated care’ is another case study used to illustrate how care coordination can be organised by a primary care doctor.^19^ The case discussions that follow help students to understand care coordination and enable them to compare that to care fragmentation. In the case of fragmented care, students are guided to propose how fragmentation could have been avoided to achieve better care outcomes.

## Reflections

From the experience of the author, the audio-visual aids are able to capture students’ attention. They make it easier to convey the concepts and principles of family medicine. The videos used are intended to summarise the didactic lectures delivered and consolidate the information received from the lecturing material more simply. Furthermore, the use of case studies is a way of identifying and implementing strategies that will give medical students both an academic foundation and practical experience.

## Conclusion

The early introduction of family medicine concepts to medical students is necessary to address the inadequacies in understanding the field. Students’ understanding can be enhanced through employing innovative teaching methods like the use of audio-visual aids and case study methods. The next steps would involve researching the impact of these teaching and learning aids in improving the understanding of students in the field of family medicine.
